# Stem and Leaf Anatomy of *Aragoa* (Plantaginaceae): In Search of Lost Rays

**DOI:** 10.3390/plants10091773

**Published:** 2021-08-26

**Authors:** Alexei Oskolski, Nathi Vuza, Alexey Shipunov

**Affiliations:** 1 Department of Botany and Plant Biotechnology, University of Johannesburg, Auckland Park 2006, Johannesburg P.O. Box 524, South Africa; msvuza@gmail.com; 2 Komarov Botanical Institute, Prof. Popov Str. 2, 197376 St. Petersburg, Russia; 3 The Kyoto University Museum, Kyoto University, Kyoto 606-8317, Japan; dactylorhiza@gmail.com

**Keywords:** rayless wood, secondary phloem, stomata, trichomes, procambium, aerenchyma, páramo, *Plantago*

## Abstract

*Aragoa* is a shrubby genus endemic to páramo in the northern Andes representing the sister group to *Plantago* and *Limosella*. Stem and leaf structure of *Aragoa corrugatifolia* were studied to clarify the evolutionary pathways and ecological significance of their anatomical traits. *Aragoa* and *Plantago* share a non-fascicular primary vascular system, rayless wood and secondary phloem, and anomocytic stomata. *Aragoa* is distinctive from most Plantaginaceae in the presence of cortical aerenchyma and of helical thickenings in vessels. Its procambium emerges in the primary meristem ring as a continuous cylinder. The view on the ring meristem and procambial strands as developmental stages in the formation of a primary vascular system is not relevant for *Aragoa*, and probably for other Plantaginaceae. The raylessness is synapomorphic for the crown clade of Plantaginaceae comprising *Aragoa*, *Littorella*, *Plantago*, *Veronica*, *Picrorhiza*, *Wulfenia*, and *Veronicastrum*. The loss of rays is thought to be predetermined by procambium rather than by the vascular cambium. The extremely narrow vessels with helical thickenings are presumably adaptive to hydric and thermic conditions of páramo. Cortical aerenchyma is thought to be a response to the local hypoxia caused by the water retained by ericoid leaves. Trichomes on juvenile leaves are expected to be the traits of considerable taxonomic importance.

## 1. Introduction

*Aragoa* Kunth is the genus with approximately 19 species endemic to the alpine páramo biome in the northern Andes of Colombia and Venezuela. The species of *Aragoa* are shrubs and small trees up to 5–8 m tall, with needle-like or scale-bar leaves and reduced axillar racemose inflorescences bearing actinomophic flowers with four white petals [1,2,3,4,5]. *Aragoa* was been considered as a member of Scrophulariaceae until the polyphyly of this family was strongly confirmed by molecular phylogenetic data. After the revision of Scrophulariaceae with its subdivision into several monophyletic groups, this genus was placed in to a vastly enlarged family of Plantaginaceae [6]. A molecular analysis based on *rbc*L and ITS sequences showed that *Aragoa* is a sister group to a large cosmopolitic genus *Plantago* of about 200 species [7]. Close relationships between these two genera were confirmed by essential similarities in morphology of their inflorescences and flowers [4]. Recent phylogenomic analyses based on plastome sequences data [8] established, however, that *Aragoa* is sister to a broader lineage comprising *Plantago* and *Littorella* (L.) Asch., a disjunct genus with three amphibious species [9].

Although the gross morphology of the *Aragoa* species has been comprehensively described by [2,3], the anatomical data on this genus remain very poor. A few details of its wood structure were mentioned in [10]; the microphotos of wood sections of four *Aragoa* species are also presented in the InsideWood database [11]. Lersten and Curtis [12] also reported the absence of idioblasts in leaves of *Aragoa dugandii* Romero. No data on bark and leaf structure of *Aragoa* are available to date. The examination of vegetative anatomy of this genus could be very important, however, for clarification of the patterns of structural trait evolution within its sister genus *Plantago* and within the Plantaginaceae family as well.

The rayless wood is a prominent feature of *Aragoa* [10]. This uncommon wood trait is also known in the majority of *Plantago* species as well as in some other members of Plantaginaceae [13,14,15,16,17,18,19,20,21]. The loss of rays can be contingent on different functional, adaptive, and/or morphogenetic factors; particularly, it may be considered as an indicator of secondary woodiness, i.e., the origin of woody habit from herbaceous ancestor [16]. Such explanation sounds plausible for mostly herbaceous Plantaginaceae. The evolution of the gains and losses of rays has not been analysed yet for this group and for any other plant family as well. 

According to [15,16,22], the raylessness in Plantaginaceae can also be considered as a manifestation of wood paedomorphosis, or protracted juvenilism. Following this theory, the rays can be lost due to retaining their absence in juvenile primary xylem at the adult developmental stage, i.e., at the secondary xylem formed by vascular cambium. Moreover, the available data (e.g., [14,17,18,19,23,24,25,26,27,28,29]) show that the members of Plantaginaceae have a continuous procambium cylinder that gradually goes into the vascular cambium. These data are in contrast to a common view on the formation of a vascular system, supposing the initiation of procambium as separated strands, giving rise to discrete vascular bundles and the formation of vascular cambium from fascicular and interfascicular cambia at the later stage of shoot development (e.g., [30,31]). At the same time, the procambial ring can contain the ray initials, as it has been reported in many plant groups [32,33,34,35,36] contrarily to a widely held view that the formation of rays is confined to secondary tissues [30]. These data suggest that the presence or absence of ray initials in procambial ring can predetermine their occurrence in vascular cambium and, therefore, it can facilitate the paedomorphic retaining of ray formation or raylessness in secondary tissues. This hypothesis on the role of procambial ring in the formation of rayless wood can be tested by the examination of development of procambium, cambium, and vascular system in a rayless taxon, such as *Aragoa*.

In the present study, we explore the stem and leaf structure of *Aragoa corrugatifolia* to clarify the evolutionary pathways and ecological significance of raylessness and other anatomical traits found in this genus.

## 2. Results

### 2.1. Development of Meristematic and Conductive Tissues in Stem

**Figure 1:**
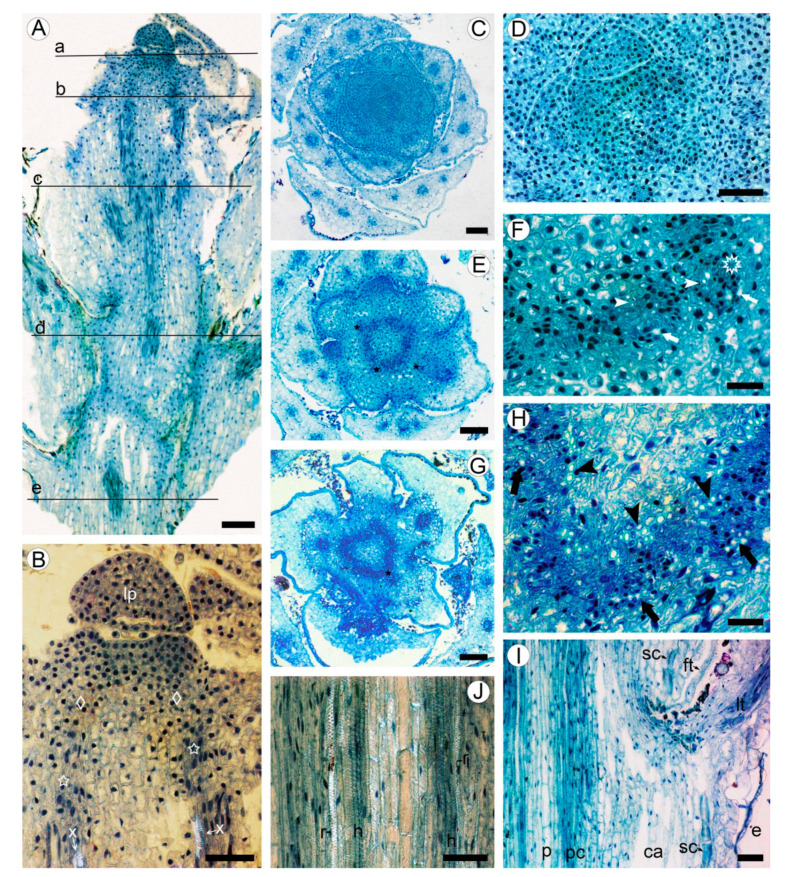
Apical meristem and primary tissues in young shoot of *Aragoa corrugatifolia*, light microscopy (LM). (**A**) Radial longitudinal section (RLS) of young shoot, approximate positions of the transverse sections (TS) shown on Figure 1C,D (a), Figure 1E,F (b), Figure 1G,H (c), Figure 11I,J (d), and Figure 2A,B (e); (**B**) tip of young shoot observed in partially polarized light (RLS), shoot apical meristem (SAM) with leaf primordium (lp) and peripheral zone (diamonds), procambium cells (stars), protoxylem elements (x); (**C**) shoot tip at the SAM level (TS), 3/8 phyllotaxis, leaf primordia with solitary leaf traces, young leaves with three vascular bundles; (**D**) SAM with leaf primordia (TS); (**E**) shoot tip just below the SAM level (TS); procambium ring, leaf traces (asterisks); (**F**) portion of the procambium ring shown on Figure 1E (TS), leaf trace (asterisk), protoxylem (arrowheads), and protophloem (arrows) elements; (**G**) young shoot in the lower portion of first elongated internode (TS), procambium ring, offset of leaf trace (asterisk). (**H**) Portion of the procambium ring shown on Figure 1G (TS), radial seriations of procambial cells, elements of primary xylem (arrowheads) and primary phloem (arrows); (**I**) stem and leaf base at the level of fifth elongated internode (RLS), procambium ring (pc), pith (p), cortical aerenchyma (ca), sclereids at the leaf base (sc), epidermis, leaf trace (lt), filiform trichome (ft); (**J**) portion of the procambium ring shown in Figure 1I observed in partially polarized light (RLS), tracheary elements with helical (h) and reticulate (r) patterns of secondary cell wall. Scale bars: 100 µm for (**A**,**C**,**E**,**G**), 50 µm for (**B**,**D**,**I**,**J**), 20 µm for (**F**,**H**).

Shoot apical meristem (SAM) is about 200 µm in diameter, surrounded with the leaf primordia and small leaves arranged with the 3/8 phyllotaxis (Figure 1A,B). The procambium is initiated in the peripheral zone of SAM (primary meristem) near the shoot tip. The procambial strands are hardly distinguished, forming nearly continuous ring of four to six cells wide, occasionally interrupted by the offsets of leaf traces (Figure 1A–D). The procambial cells are angular isodiametric (6–9 µm in width) in transverse view, and elongated (26–48 µm in length) on longitudinal sections. Differentiation of the protoxylem and protophloem begins almost immediately with the formation of groups of one to two helical tracheary elements and one to three sieve elements (Figure 1E,F).

At the level of the base of the first elongated internode (ca. 0.4 mm from the apex), the procambium ring widens up to 7–10 cells wide, interrupted only by the gaps at the offsets of leaf traces (Figure 1G,H). The procambial cells in the ring show a tendency for radial seriation; the sites of leaf trace initiation can be distinguished as the clusters of cells divided at various orientations. The elements of primary phloem are in groups of two to four scattered along the outer side of the procambium ring. These tracheary elements are solitary and grouped as two to five cells, found in the inner region of procambium ring, without obvious association with the groups of phloem elements. The proto- and metaxylem consists of tracheary elements with helical and reticulate patterns of secondary cell walls.

**Figure 2:**
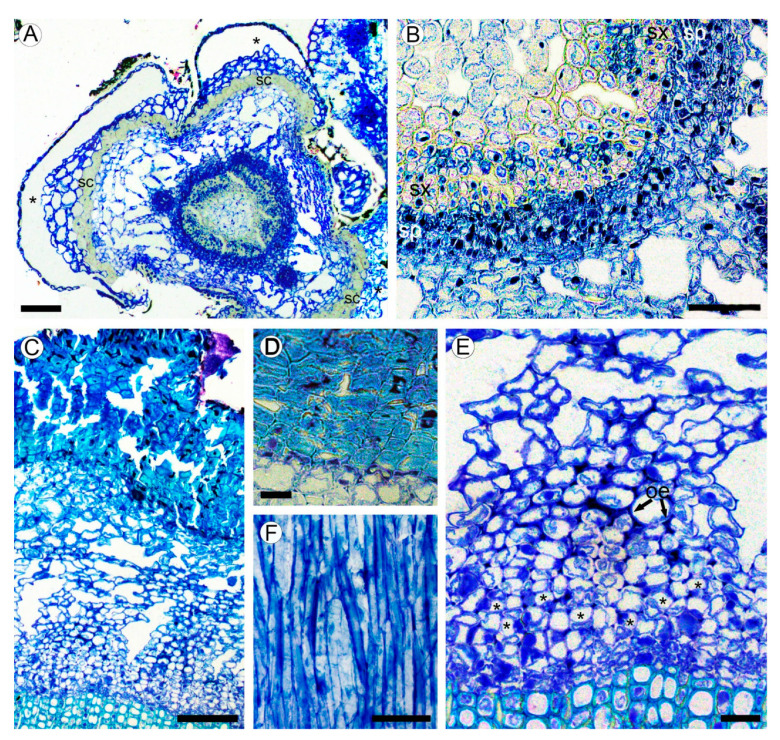
(**A**,**B**) Structure of young stem in *Aragoa corrugatifolia* (LM). (**A**) Young shoot with prominent leaf bases in the lower portion of fourth elongated internode (TS), vascular cambium with juvenile rings of secondary xylem and secondary phloem, cortical aerenchyma, leaf bases (asterisks) outlined with the bands of sclereids (sc); (**B**) portion of the ring of secondary conductive tissues shown in Figure 2A (TS), continuous rings of secondary xylem (sx) and secondary phloem (sp). (**C**–**F**) Structure of mature bark. (**C**) Secondary phloem, cortex with aerenchyma, periderm with uniseriate phelloderm, and prominent phellem made of thick-walled cell (TS); (**D**) Portion of periderm, phellem cells with thick sclerified walls, thin-walled phelloderm cells in one to two layers (TS); (**E**) portion of secondary phloem and cortical aerenchyma shown in Figure 2C (TS), sieve tubes with companion cells in radial groups (*), obliterated phloem elements (oe), large intercellular spaces in cortex; (**F**) tangential longitudinal section (TLS) of secondary phloem; strands of phloem axial parenchyma, lack of rays. Scale bars: 100 µm for (**A**,**C**), 50 µm for (**B**,**F**), 20 µm for (**D**,**E**).

In the lower internodes, the vertical length of procambium cells increases due to the shoot growth up to 35–67 µm in the fifth internode (Figure 1I,J). These cells are more tapered than those near the shoot tip. Some cells undergo transverse divisions, forming the strands of two cells. No short procambium cells resembling the ray initials were found. Below the sixth internode, the procambium ring is gradually transformed into the vascular cambium, which is recognized by the appearance of the continuous rings of regularly arranged phloem and xylem elements around the pith made of thin-walled isodiametric parenchyma cells of 15–25 µm, with large intercellular spaces in between. Sclereids with moderately thick cell walls were found in the outermost region of pith (Figure 2A,B).

### 2.2. Bark Structure

A large portion of the circumference of young stems is occupied by the bases of leaves covered with the leaf abaxial epidermis. The zones of attachment of leaf bases to the stem cortex are outlined with the uniseriate (occasionally biseriate) layers of thick-walled vertically elongated sclereids of 18–34 µm in tangential size and 32–54 µm in vertical size (Figure 1J and Figure 2A). Between the leaf bases, the epidermis on young parts of stems is composed of a single layer of isodiametric cells (15–25 µm in tangential size) with evenly thick walls (3–7 µm thick) covered by a prominent cuticle that is 8–10 µm thick, with dark deposits. The cortex is narrow (10–12 cells in width) and composed of aerenchyma made isodiametric to elongated thin-walled parenchyma cells of 15–35 µm in size, with large intercellular spaces in-between. No crystals were found in the cortical parenchyma cells. Primary phloem fibers were absent (Figure 2A,B). Dilation of the cortical tissue is effected mostly by tangential stretching of parenchyma cells and intercellular spaces.

Mature bark is dark brown, non-peeling, without fissures and scales. The initiation of periderm is in the subepidermal layer of cells. The phellem is composed of 8–15 layers isodiametric to somewhat flattened cells with thick to very-thick sclerified cell wall occasionally with dark deposits. The phelloderm comprises one to two layers of isodiametric, thin-walled cells. No crystalliferous cells were found in periderm. Subsequent periderms were not found (Figure 2C,D).

Sieve tubes are in radial groups of two to seven. Sieve tubes members are 148–236 µm (average 184.2 µm) in length and are 9–18 µm wide. Sieve plates are compounded with two to three sieve areas, located on vertical or slightly oblique cross walls. Axial parenchyma cells are associated with conducting elements that occur as single fusiform cells and in strands of two to three cells. The transition from conducting to nonconducting secondary phloem is gradual, marked with obliterated conductive cells and slightly tangentially stretched axial parenchyma cells (Figure 2E). Secondary phloem rays were not found (Figure 2F).

### 2.3. Leaf Structure

**Figure 3:**
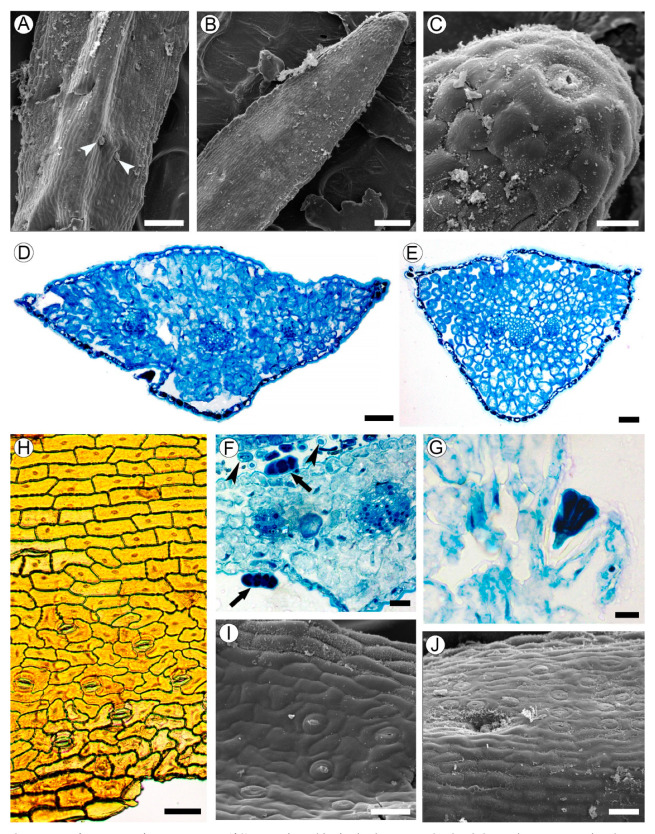
Leaf structure of *Aragoa corrugatifolia.* (**A**) Abaxial leaf side (SEM), sessile glandular trichomes (arrowheads); (**B**) adaxial leaf side (SEM), hydathode pore on the leaf tip; (**C**) hydathode pore at the leaf tip shown on Figure 3B (SEM); (**D**) TS of leaf in its middle portion (LM), epidermis with prominent cuticle, spongy mesophyll, three vascular bundles; (**E**) TS of leaf in its basal portion (LM), epidermis with prominent cuticle, spongy mesophyll, branch point of vascular bundles; (**F**) fragment of leaf (TS, LM), sessile glandular trichomes on adaxial and abaxial epidermis (arrows), cross sections of filiform trichomes (arrowheads); (**G**) stalked glandular trichome on adaxial epidermis (LM); (**H**) Abaxial epidermis (LM) from midrib without stomata (top) and lateral portions of leaf with anomocytic stomata (bottom). (**I**) Adaxial leaf side (SEM), anomocytic stomata, epidermal cells with curved anticlinal walls. (**J**) Midrib region on adaxial leaf side (SEM), epidermal cells with straight anticlinal walls, hollow with glandular trichome. Scale bars: 200 µm for (**A**,**B**), 50 µm for (**C**–**E**,**H**–**J**), 20 µm for (**F**,**G**).

Leaves are linear, 1–2 mm in width, sessile, with convex abaxial side, and flat adaxial surface, and are 200–400 µm thick. The adaxial epidermal cells do not differ from the abaxial ones in their shape and size; these cells are flattened to isodiametric, 18–30 µm in tangential size, with even walls of approximately 1 µm thick (Figure 3A,B). Hydathode pores are present at the leaf tips (Figure 3C).

Leaf blades are isobilateral (Figure 3D,E). The spongy mesophyll consists of irregularly shaped cells of 11–25 µm in size with large intercellular spaces. The vascular bundles are collateral, sheathed by one or two layers of parenchyma cells without lateral or vertical extensions. Each leaf is supplied by a single trace that is forked into three vascular bundles near the leaf base.

Long (3–4 mm in length) multicellular filiform trichomes are found at the bases of juvenile leaves on their adaxial sides, and usually lack mature leaves (Figure 1I and Figure 3F). Glandular trichomes sessile or on unicellular stalks, with four head cells, are located mostly in hollows sparsely scattered on both leaf sides (Figure 3A,F,G,J).

Anticlinal walls of epidermal cells are mostly straight in the midrib region, or mostly curved outside of it. The outer walls of the epidermal cells are covered by a cuticle of approximately 4–7 µm thick. Leaves are amphistomatic; stomata are more numerous on the abaxial side (48–62 per mm^2^) than on the adaxial side (39–50 per mm^2^), lacking in the midrib region. Stomata anomocytic is located in the same plane with epidermal cells (Figure 3H–J).

### 2.4. Wood Structure

**Figure 4:**
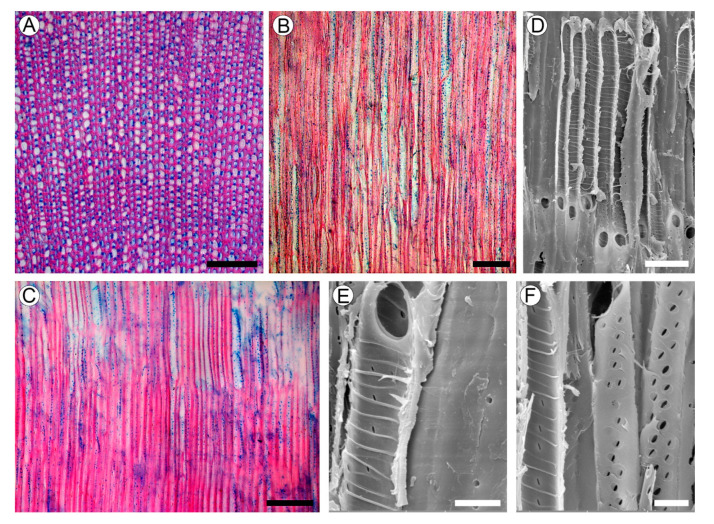
Wood structure of *Aragoa corrugatifolia*. (**A**) TS of wood (LM), growth rings absent, very narrow vessels, libriform fibers with living protoplasts; axial parenchyma and rays absent; (**B**) TLS of wood (LM), very narrow vessels, lack of rays; (**C**) RLS of wood (LM), imperforate elements with numerous pits in radial walls, lack of rays; (**D**) vessel elements on RLS (SEM), simple perforation plates, helical thickenings on vessel walls; (**E**) vessel element and fibriform fiber on RLS (SEM), simple perforation plates, helical thickenings on vessel walls, pits on the fiber wall (right); (**F**) vessel elements on RLS (SEM), helical thickenings on vessel walls; minute intervessel pits. Scale bars: 100 µm for (**A**–**C**), 10 µm for (**D**–**F**).

Growth rings absent. Wood is rayless (Figure 4A–C). Vessels are angular; rarely rounded in outline; extremely narrow with a tangential diameter of 9.2–19 µm (average 13.5 µm); and are extremely numerous (1130–1700 per mm^2^), mostly solitary, and also has multiples. Vessel walls are 1.6–3.4 µm thick. Tyloses were not found. Vessel elements are 102–275 µm long (average 202.6 µm). Perforation plates are simple. Intervessel pits are alternate and minute, 1.8–3.5 µm in vertical size, mostly with rounded margins and slit-like apertures. Helical thickenings throughout the body of the vessel element are common (Figure 4D–F). Ground tissue elements are non-septate libriform fibers of 190–317 µm (average 255.2 µm) in length, mostly with living protoplast and nuclei. Fiber walls are 2.0–5.4 µm thick, with distinctly bordered pits of 2.4–3.5 µm in vertical size, with slit-like apertures in radial and tangential walls. Axial parenchyma was not observed.

### 2.5. Evolution of Rayless Wood within Plantaginaceae

**Figure 5:**
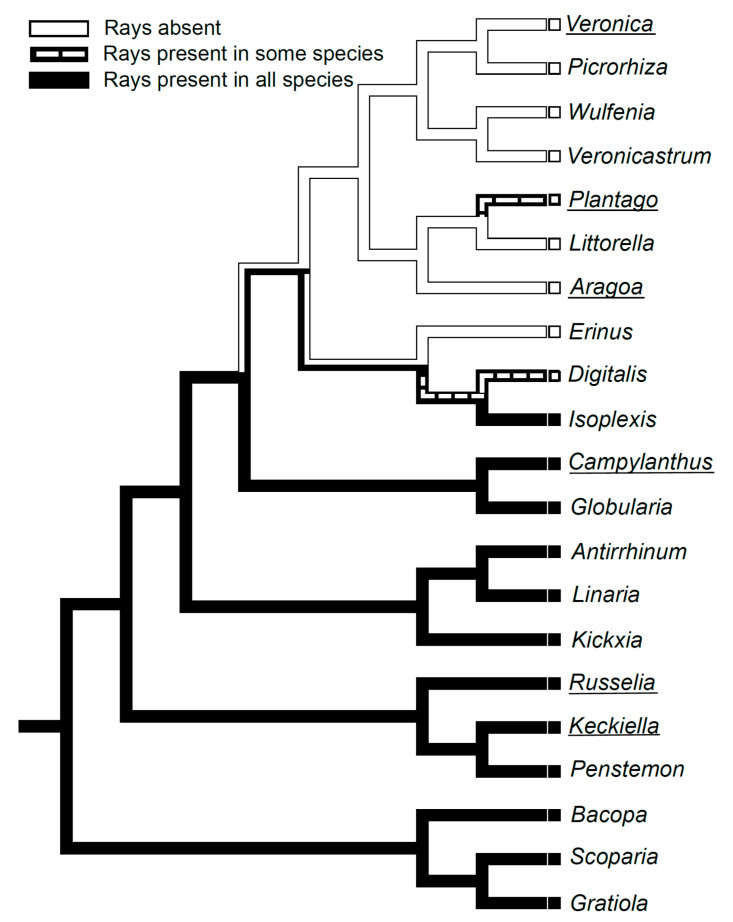
Distribution of the presence or absence of rays in the wood mapped onto a subsample of the most parsimonious tree of the combined analysis of one nuclear and three plastid regions for the family Plantaginaceae [8]. The genus *Littorella* was added to this tree following [9]. The names of genera containing shrubby species are underlined; other genera are herbaceous.

Both our observations on *Aragoa*, and the published wood anatomical data on other Plantaginaceae genera [11,13,14,15,17,18,19,20,21,27,29,37,38], were used to reconstruct the pattern of evolution for the raylessness within this family (Figure 5). The mapping of this trait on a subset of the most parsimonious tree of the combined analysis of one nuclear and three plastid regions [6] shows that the loss of rays is synapomorphic for the large crown clade comprising the genera *Aragoa*, *Littorella*, *Plantago*, *Veronica*, *Picrorhiza*, *Wulfenia*, and *Veronicastrum*, and it occurred twice independently in the genera *Erinus* and *Digitalis*, belonging to its sister lineage.

## 3. Discussion

*Aragoa corrugatifolia* is similar to its closely related genus *Plantago* as well as to other Plantaginaceae genera in that it has a non-fascicular primary vascular system, rayless wood and secondary phloem, simple perforation plates, lack of axial parenchyma in wood, the homogenous secondary phloem lacks sclereids and crystalliferous cells, and the isobilateral amphistomatic leaves lacks anomocytic stomata [11,14,15,17,18,19,39,40]. At the same time, *Aragoa* is distinctive from most Plantaginaceae in the presence of aerenchyma in the cortex and of helical thickenings on the vessel walls. Within this family, the aerenchyma was also found in stems of *Bacopa*, *Gratiola*, *Littorella*, and one herbaceous species of *Veronica* [18,19,27,38], whereas the helical thickenings were reported in *Kekiella* [37] and in two shrubby species of *Veronica* (sect. *Hebe*) [17]. The combination of these traits may be presumed as diagnostic for this genus.

The organization of procambium and primary vascular system in the stem of *A. corrugatifolia* is nearly the same as in *Veronica speciosa* R. Cunn. ex A. Cunn. (=*Hebe speciosa* (R. Cunn. ex A. Cunn.) Andersen), another member of Plantaginaceae [23,25]. The procambium in *Aragoa* stem emerges in the primary meristem ring as a continuous cylinder encompassing the leaf traces at different stages of their formation. This procambial cylinder is interrupted only by the gaps at the offsets of leaf traces, without separation into individual strands. The fascicular pattern of the primary vascular system in *Aragoa* is manifested in the arrangement of few xylem and phloem elements separated by wide continuous rings of meristematic cells. This procambial ring entirely continues into the vascular cambium, recognized only by the formation of secondary xylem and secondary phloem. The presence of the continuous ring of vascular tissues in juvenile stems of all other members of this family examined to date (e.g., [14,17,18,19,23,24,25,26,27,28,29]) strongly suggests that such an organization of primary plant bodies is characteristic for the entire family.

Our data confirm, therefore, that the meristematic cylinder observed in young stems of *Aragoa* and *Veronica* is not confined to the primary meristem ring, i.e., to the peripheral region of SAM extended along the stem. Instead, it is formed by the coalescence of procambial strands that are initiated at the primary meristem [25,41]. This meristematic cylinder comprises both procambium and primary meristem, but these two components are practically indistinguishable from each other. These data strongly suggest that the commonly accepted view on the ring meristem and procambial strands as different developmental stages of the same gradual process of formation of primary vascular system (e.g., [31]) is not relevant for *Aragoa*, *Veronica*, and probably for other Plantaginaceae. In *Aragoa*, the separate strands of procambium can be distinguished only just beneath the SAM, forming entire procambial rings in lower portions of the stem. Apparently, the shifts from continuous procambium ring to separate strands occur in some *Plantago* species (*P. albicans*, *P. webbii*) that have broad medullary rays in their wood [18]. A detailed examination of early development of shoots in these species could be important to clarify the ways of evolutionary transitions between fascicular and non-fascicular patterns of procambium and primary vascular systems.

Our data confirm the absence of rays in wood of the *Aragoa* reported by [10], and in its secondary phloem as well. The mapping on the phylogenetic tree showed that the raylessness is synapomorphic for the large crown clade within Plantaginaceae comprising the genera *Aragoa*, *Littorella, Plantago*, *Veronica*, *Picrorhiza*, *Wulfenia*, and *Veronicastrum*. This trait also occurred in the genera *Erinus* and *Digitalis*, belonging to the sister lineage of this clade [11,13,14,15,17,18,19,20,21,27,29,38]. As the rayless species of Plantaginaceae occur in very contrasting habitats ranging from springs, bogs, and tidal zones, to arid and alpine biomes, showing a variety of life forms from annual herbs to shrubs and small trees, the loss of rays is hard to explain in terms of their functional or adaptive value [42]. Although the position of rayless woody taxa (*Aragoa*, some *Plantago* and *Veronica*) on phylogenetic trees [6,43] strongly suggest their origin from herbaceous ancestors, the consideration of the raylessness as anatomical evidence for derived woodiness in these particular genera ([5] following [44]) is highly questionable, at least for the Plantaginaceae.

In the plants with procambial rings, the presence of rays as well as their absence is displayed from the very initiation of continuous rings of conductive tissues [26]. In Plantaginaceae, the microphotos of cross sections of the stem in *Bacopa monnieri* [27] show that the rays in its wood are initiated from the narrow (one to two seriate) portions of the ring of vascular tissues located between regularly arranged radial files of proto- and metaxylem elements. In the rayless *Aragoa*, however, the files of primary xylem are widely spaced, and no traits of ray formation have been observed in the procambium ring. These data allow us to speculate that the formation of rays or their loss is predetermined by the procambium rather than by the vascular cambium. If that is so, then the raylessness in *Aragoa* and in other Plantaginaceae occurred as a result of the lack of rays initials in their vascular cambium that are retained from the procambial ring. In this case, the raylessness in this plant group may be considered as a paedomorphic feature in the proper sense of this term. Unlike Sh. Carlquist’s theory of paedomorphosis [15,22], however, the retaining of this juvenile (procambial) trait at the adult stage of secondary growth is not necessarily associated with the shift from herbaceous to woody habit. Detailed studies of the ray initiation in other groups of Plantaginaceae are required to test this hypothesis.

The extremely narrow vessels (<20 µm in diameter) with helical thickenings on their inner walls found in *Aragoa* are presumably adaptive to hydric and thermic conditions of páramo. The predominant climate of this biome is characterized by rainfall throughout the year with extreme daily temperature variations and frequent high air evaporative demands. Although the plants of this biome live under permanent rains, drizzle, and fog, they undergo regular water stress and occasional freezing [45,46,47]. In *Aragoa*, the small vessel diameter and the presence of helical thickenings on their inner walls found are thought to be the traits facilitating the tolerance of the hydraulic system to water stress by decreasing the risk of vessel blockage by air embolism [44,48,49,50,51]. Higher resistance of narrow vessels to embolism than of wide ones has been confirmed experimentally [52,53,54,55,56]. The presence of helical thickenings is also associated with the regions that experience water stress [44,51], but their functional role remains obscure [57].

The absence of axial parenchyma found in *Aragoa* is also characteristic for most other rayless members of Plantaginaceae with few species of *Plantago* and *Veronica* as exceptions [17]. The opposite combination of the lack of axial parenchyma with the presence of rays is also uncommon in this family being reported only in *Campylanthus* and few *Kickxia* species [18]. This correlation between the raylessness and the lack of axial parenchyma noted by [16,42] supports an idea of close functional and morphogenetic connectivity between those two parenchymatous components of wood [58]. Obviously, the functions of lacking parenchyma in the wood of *Aragoa* [59] are performed by the fibers with living protoplasts interconnected by large pits on their radial walls. The presence of such fibers has been reported also in some *Plantago*, *Veronica*, and *Wulfenia* species [15,18].

The presence of aerenchyma in the cortex of the stem in *Aragoa* is also noteworthy. Its formation is commonly associated with hypoxia resulting from waterlogging, but it may also be caused by other forms of stress [60]. Among Plantaginaceae, the aerenchyma was found either in submersed aquatic plant *Littorella uniflora* (L.) Asch., or in riparian species *Veronica beccabunga*, *Bacopa monnieri*, and *Gratiola officinalis* [18,19,27,38] that are also subjected to flooding. Although *Aragoa corrugatifolia* also grows in moist habitats, it does not suffer from waterlogging. We can speculate that its aerenchyma is formed in response to the local hypoxia caused by the water retained by densely arranged ericoid leaves on the young shoots. Special research is required to test this hypothesis.

The leaves of *Aragoa corrugatifolia* show a combination of xeromorphic (prominent cuticle) and mesomorphic (hydathodes) traits that are consistent with the ever-changing páramo environment. While the presence of glandular and nonglandular trichomes on leaves has long been considered as an important trait for systematics of the genus *Plantago* [40,41,61,62,63], the leaf indumentum of *Aragoa* has never been properly described. In his taxonomic treatment of this genus, Fernández-Alonso [3] mentioned only the presence of ciliate trichomes at the leaf margins as a characteristic trait of the section Ciliateae comprising *A. kogiorum*, *A. lucidula*, and *A. funkii*. We found that sessile glandular trichomes and filiform non-glandular occur on juvenile leaves of *A. corrugatifolia* belonging to the type section of this genus, but those are lost on mature leaves. Our observations suggest that the indumentum of young leaves in other *Aragoa* species also merits attention due to its potential taxonomic importance.

## 4. Materials and Methods

The sample of *Aragoa corrugatifolia* was collected by the third author and Ekaterina Shipunova on 16 March 2017 on páramo Sumapaz (west of the road in small ravine). Herbarium voucher (Shipunov P-1283) was deposited in COL. The pieces of stem and branches with leaves were fixed in 70% ethanol.

The pieces for wood anatomical study were taken from the thickest portion of the stem of this plant individual. Transverse, radial, and tangential sections (thickness 20–25 µm) were made on rotary microtomes (Ernst Leitz GMBH, Wetzlar, Germany and Jung AG Heidelberg, Germany) and stained with a 1:1 alcian blue/safranin mixture. Macerations were made using Jeffrey’s solution [64]. Fifty measurements for each quantitative wood anatomical trait were made on sections and macerated wood.

The specimens for scanning electron microscopy were prepared according to the methods of [65], including cutting with safe razors into the sections of ca. 1 mm in thickness, drying in open air in Petri dishes covered by paper to protect the samples from dust, mounting on stubs with double-stick tape, and coating with gold. The samples were investigated at 10 kV with working distance 8 mm with the TESCAN microscope (TESCAN, Brno, Czech Republic) using the VegaTS software at the central analytical facility (Spectrum) of the University of Johannesburg. Descriptive terminology for the wood structure follows recommendations of the [39] except for the recording of the vertical diameter of intervessel pits, as this trait is more suitable for comparative studies due to its lesser variability than horizontal pit size recommended by the IAWA Committee [66].

The evolution of raylessness in Plantaginaceae was clarified by mapping this trait onto a subsample of the most parsimonious tree of the combined analysis of one nuclear and three plastid regions for the family Plantaginaceae [6]. The genus *Littorella* was added to this tree following [8]. Character optimization along the branches of the tree was illustrated using the parsimony reconstruction method of the Character History Tracing option in the computer package Mesquite 2.75 [67].

The structure of juvenile stems and mature bark was examined on the samples embedded in glycol methacrylate (GMA) according to a modification of the [68] method. Transverse, tangential, and radial sections of about 5 µm thick were cut by using a Porter-Blum MT-1 ultramicrotome. Bark sections were then stained with toluidine blue method before being mounted in Entellan. Measurements of sieve tube members were made in fragments of secondary phloem macerated using Jeffrey’s solution [37]. For examination of sieve plate morphology, the radial sections of mature bark of 20–25 µm were made with freezing microtome (Ernst Leitz GMBH, Wetzlar, Germany), stained with resorcin blue according to [69], and embedded in Euparal. Descriptive bark anatomical terminology follows [70].

## Data Availability

Data is contained within the article.
